# On the Accuracy Potential in Underwater/Multimedia Photogrammetry

**DOI:** 10.3390/s150818140

**Published:** 2015-07-24

**Authors:** Hans-Gerd Maas

**Affiliations:** TU Dresden, Institute of Photogrammetry and Remote Sensing, Helmholtzstr. 10, D-01069 Dresden, Germany; E-Mail: hans-gerd.maas@tu-dresden.de; Tel.: +49-351-46332859

**Keywords:** underwater photogrammetry, multimedia photogrammetry, geometric modeling, accuracy analysis

## Abstract

Underwater applications of photogrammetric measurement techniques usually need to deal with multimedia photogrammetry aspects, which are characterized by the necessity of handling optical rays that are refracted at interfaces between optical media with different refractive indices according to Snell’s Law. This so-called multimedia geometry has to be incorporated into geometric models in order to achieve correct measurement results. The paper shows a flexible yet strict geometric model for the handling of refraction effects on the optical path, which can be implemented as a module into photogrammetric standard tools such as spatial resection, spatial intersection, bundle adjustment or epipolar line computation. The module is especially well suited for applications, where an object in water is observed by cameras in air through one or more planar glass interfaces, as it allows for some simplifications here. In the second part of the paper, several aspects, which are relevant for an assessment of the accuracy potential in underwater/multimedia photogrammetry, are discussed. These aspects include network geometry and interface planarity issues as well as effects caused by refractive index variations and dispersion and diffusion under water. All these factors contribute to a rather significant degradation of the geometric accuracy potential in underwater/multimedia photogrammetry. In practical experiments, a degradation of the quality of results by a factor two could be determined under relatively favorable conditions.

## 1. Introduction

Photogrammetric tasks requiring the tracing of optical rays through multiple optical media with different refractive indices are called “multimedia photogrammetry” in the photogrammetric literature. This denomination has already been coined at a time, when the term “multimedia” was completely unknown in its contemporary meaning of the combined use of digital media such as text, images, film, animation and audio.

Multimedia photogrammetry is characterized by the refraction of optical rays at the transition between optical media with different refractive indices, which can be modeled by Snell’s Law. An early treatment of this issue can be found in [1], who worked on the relative orientation of stereo aerial images of underwater scenes on analogue plotters and coined the term “two-media photogrammetry”. References [2,3] showed an analytical solution and took the step from two- to multimedia photogrammetry, replacing the straight imaging rays by polygons. Kotowski [4] developed a ray-tracing method for tracing rays through an arbitrary number of parameterized interfaces, which was implemented in a bundle adjustment. It allows for handling both image invariant and object invariant interfaces.

In photogrammetry, we can distinguish three major categories of applications of multimedia techniques:

- In aerial photogrammetry, photo bathymetry is a technique to derive models of the sea floor from stereo imagery, provided limited depth and sufficient water transparency [3,5]. The air-water transition can be modeled on the basis of Snell’s Law. Most implementations herein assume the water surface to be horizontal and planar, with waves on the water surface leading to significant errors [6].
- In underwater photogrammetry, cameras (with suitable housing) are used underwater. Some of these cameras are equipped with lenses specially designed for underwater imaging. As an alternative, cameras may be equipped with a planar front window, which can geometrically be treated as an image invariant interface. Typical application examples are in archaeology [7], the recording of ship wrecks, marine biology [8], measurements in nuclear power stations [9] or in the measurement of the shape of fishing nets [10].
- Many applications in industrial/technical close range photogrammetry deal with objects or processes in liquids, which are observed by cameras situated outside the observation vessel, imaging the scene through a planar window (e.g., 3D flow velocity measurement techniques [11,12]). The ray path herein is a twice-broken beam, which is refracted when passing through the three optical media interfaces air-glass-liquid (or vice versa).

Also in lidar bathymetry, which is used to determine underwater topography by airborne laser scanning [13,14], geometric models are used which can be derived from the above categories.

In the following, a multimedia model first introduced by Maas [11] will be shown, which can flexibly be integrated as a module in standard tools of photogrammetry. Subsequently, several extensions of the model will be discussed. The second part of the paper addresses several factors degrading the accuracy potential of underwater/multimedia photogrammetry.

## 2. A standard Model for Multimedia Close Range Photogrammetry

Many applications of multimedia close range photogrammetry in industrial-technical applications require the observation of objects or processes in a liquid through a glass window, which can be considered a plane parallel plate. This configuration allows for some algorithmic and computational simplifications, which form the basis for a flexible—yet strict—multimedia photogrammetry model (see also [11,15]). The model can be integrated as a module into the collinearity equations and can thus be used in photogrammetric standard procedures such as spatial resection, forward intersection, bundle adjustment or epipolar line computation. Like almost all approaches shown in the literature, the model assumes homogeneity and isotropy of the optical media.

**Figure 1:**
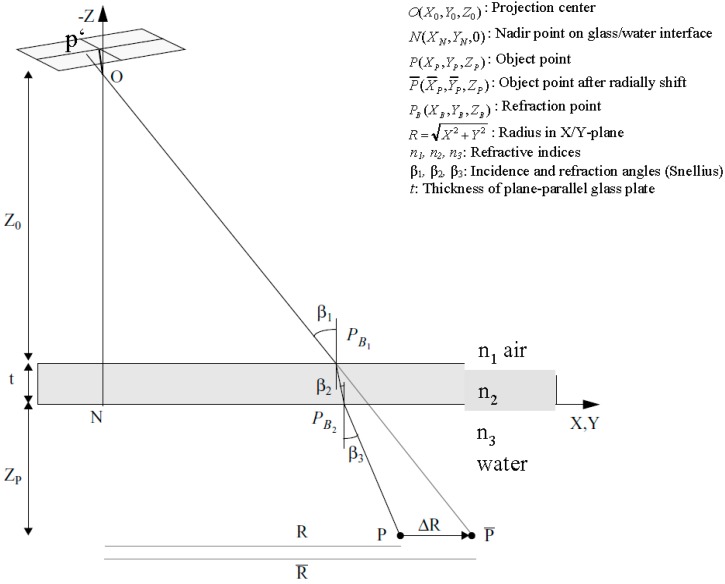
Radial shift for multimedia effect compensation [11].

The collinearity condition per definition connects image coordinates, camera projection center and object point coordinates. Its basic assumption, that image point, camera projection center and object point form a straight line, is not fulfilled any more in multimedia photogrammetry due to the refraction of the rays at the multimedia interfaces. The approach proposes a radial shift of an underwater object point with respect to the camera nadir point in a way that the collinearity condition if re-established. This radial shift is implemented as a correction term into observation equations derived from the collinearity equation. Simplifications can be achieved when defining the coordinate system in a way that the X/Y-plane is identical with one of the interface planes glass/water or air/glass (see Figure 1).

The procedure can be explained as shown in Figure 1: An object point P is imaged onto image point p′ through the water-glass and glass-air interfaces. Obeying to Snell’s Law, the imaging ray is refracted twice on its path and thus not suited for the collinearity condition. If P were radially shifted to $\bar{P}$ in a plane parallel to the X/Y-plane, the collinearity condition could be applied with $\bar{P}$ like in the standard one-media case. Therefore the goal is to compute the radial shift ∆R relative to the nadir point N  (∆R > 0 if *n*_2_ *> n*_1_ and *n*_3_ *> n*_1_). This will, for instance, allow using the radially shifted point in a spatial resection for camera orientation and calibration. Typical values might be *n*_1_*(air) = 1.0*, *n*_2_*(glass) = 1.5*, *n*_3_*(water) = 1.34* (cmp. Equation (4)).

The calculation of the radial shift ∆R can be derived from Figure 1:

(1) $$R=Z_{0}\tan{\text{β}}_{1}+t\tan{\text{β}}_{2}+Z_{P}\tan{\text{β}}_{3}$$

and

(2) $$\bar{R}=\left(Z_{0}+t+Z_{P}\right)\tan{\text{β}}_{1}$$

Snell’s Law connects the incidence angles:

(3) $$n_{1}\sin{\text{β}}_{1}=n_{2}\sin{\text{β}}_{2}=n_{3}\sin{\text{β}}_{3}$$

The thickness of the glass plate *t* and its refractive index *n*_2_ are usually assumed to be known and fixed. The refractive index of water depends on the optical wavelength as well as water temperature, salinity and depth and can be obtained from an empirical formula as used in [2]:

(4) $$n_{w}=\text{1.338}+\text{4}\times {\text{10}}^{-\text{5}}\text{(486}-\text{λ}+\text{ 0.003d}+\text{50S}-\text{T)}$$

(with *n_w_* = refractive index of water, *d* = water depth (m), λ = wave length (nm), T = water temperature (°C), S = water salinity (%)).

A closed solution of the above equation system is not possible due to the trigonometric functions. Therefore an iterative procedure is being used, wherein *P* itself is chosen as a first approximation of $\bar{P}$:

(5) $${\bar{R}}_{\left(0\right)}=\sqrt{{\left(X_{P}-X_{0}\right)}^{2}+{\left(Y_{P}-Y_{0}\right)}^{2}}$$

For the 1. iteration we get the incidence angle in medium 1 from (Equation (2))

(6) $$\beta_{1}=\arctan(\frac{{\bar{R}}_{\left(0\right)}}{Z_{0}+t+Z_{P}})$$

and subsequently the incidence and refractive angles in the other media from (Equation (3))

(7) $$\beta_{2}=\arcsin(\frac{n_{1}}{n_{2}}\sin\beta_{1})\;\beta_{3}=\arcsin(\frac{n_{1}}{n_{3}}\sin\beta_{1})$$

This yields a correction term

(8) $$\Delta R=R-(Z_{0}\tan\beta_{1}+t\tan\beta_{2}+Z_{P}\tan\beta_{3})$$

and the radial shift for the 1. Iteration

(9) $${\bar{R}}_{\left(1\right)}={\bar{R}}_{\left(0\right)}+\Delta R$$

with ${\bar{R}}_{\left(1\right)}$ we get new incidence and refractive angles β1, β2, β3, which can be used to compute a new ∆R *etc.*, until ∆R< ϵ  (e.g., with ε = 0.0001 mm).

Switching back from polar to Cartesian coordinates after the last iteration, we get the coordinates of the radially shifted point $\bar{P}$:

(10) $${\bar{X}}_{P}=X_{0}+(X_{P}-X_{0})\frac{\bar{R}}{R}$$

$${\bar{Y}}_{P}=Y_{0}+(Y_{P}-Y_{0})\frac{\bar{R}}{R}$$

$${\bar{Z}}_{P}=Z_{P}$$

$\bar{P}$ can then be used in the collinearity equation instead of P, so that the equation can be used as an observation equation in spatial resection, forward intersection (with two or more images) or bundle adjustment. This offers the great advantage that existing photogrammetric software solutions can be extended by a multimedia module handling the radial shift procedure, without any modification in the core software tools. That means that the whole multimedia problem is simply out-sourced into the radial shift computation module.

The procedure can easily be extended to an arbitrary number of parallel interfaces. It should be noted that the approach is generic with respect to the camera viewing direction and not limited to viewing directions perpendicular to a planar glass interface (as is commonly the case in underwater photogrammetry models). The model can also be deduced from the generalized model shown in [9] with the simplifications shown here. A related approach is has been shown in [16], who very vividly connects it with the “apparent places” as known from astronomical geodesy.

## 3. Computational Acceleration

As stated above, there is no closed solution to obtain the radial shift from the above equation system. There is only a ray-tracing based straight-forward solution limited to forward intersection, which avoids the procedure via the radial shift [11]. However, this solution is restricted to two cameras and does not include an adjustment, thus not making proper use of the redundant information.

The computation time in the iterative procedure for determining the radial shift parameter in the strict solution as shown in Section 2 may be reduced by about 50% by introducing an over-compensation factor [11]. A much more efficient reduction of the computational effort can be achieved by outsourcing the multimedia calculations into a lookup-table. This may for instance be relevant in photogrammetric 3D-PTV (particle tracking velocimetry) systems [17], where the coordinates of several thousand neutrally buoyant tracer particles in a liquid flow have to be determined from image sequences of three or four cameras over several seconds or minutes at 25 Hz imaging rate. In the processing of these image sequences, millions of forward intersections have to be computed, each of them requiring the iterative multimedia shift procedure. In a lookup-table based solution, the problem can be reduced to the initialization of a two-dimensional lookup-table with the depth $Z_{P}$ and the radial nadir point distance R of a point P as entry parameters and the radial shift ratio $\bar{R}/R$ as a result. Due to the reference to the nadir point, one lookup-table per camera has to be established. The lookup-table entries can be generated in a two-dimensional $\left(Z_{P},R\right)$ raster using the iterative model shown above. If the lookup-tables are initialized at a sufficient density, the relative radial shift of each point can easily be obtained by bilinear interpolation in the lookup-tables. The loss of accuracy caused by this interpolation-based procedure depends on the density of the initialization of the lookup-tables. [11] shows that less than 2000 lookup-table entries provide a good basis for handling the multimedia geometry without significant loss of accuracy in a typical 3D-PTV constellation.

## 4. Epipolar Lines in Multimedia Photogrammetry

**Figure 2:**
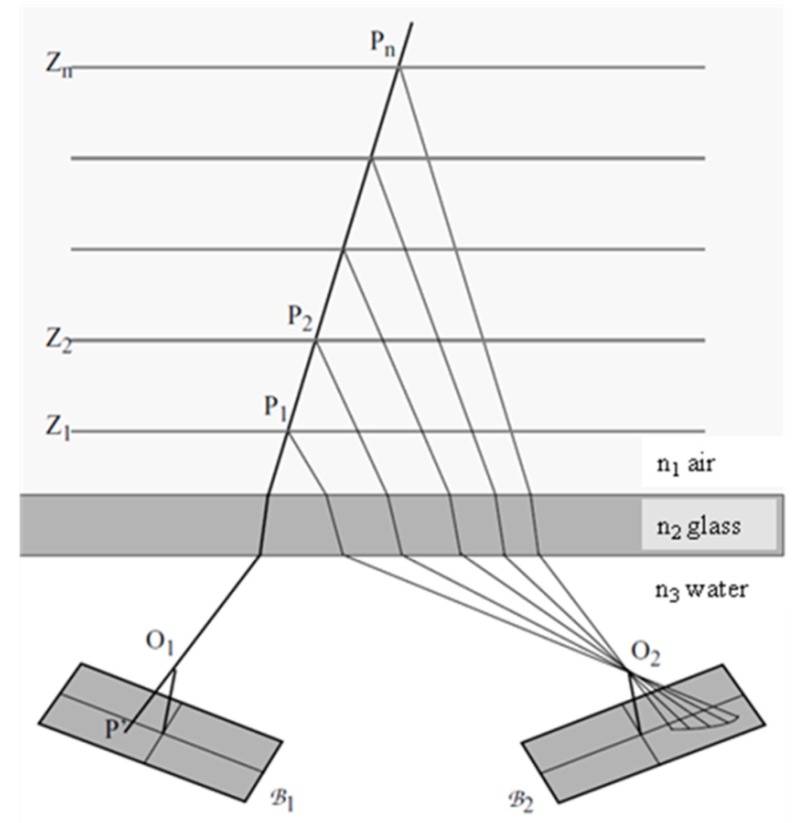
Epipolar geometry in multimedia environment.

An additional effect in multimedia photogrammetry is the fact that epipolar lines are not straight lines anymore, as can be seen from Figure 2. As a consequence, epipolar line based image matching techniques have to deal with curved epipolar lines, and the computation of rectified normal images as a pre-processing step for applying dense image matching techniques gets more complicated. The actual amount of bending depends on the proportion of the optical path lengths in air and water. While the effect may be negligible in the case of underwater photogrammetry (where the camera is oriented perpendicular to a glass window and the path length in air is short), it will usually be relevant in industrial/technical applications of close range photogrammetry, where objects or processes in liquids are observed by cameras situated outside the observation vessel. In [11], a procedure to approximate the bended epipolar line by a polygon on the basis of intersections of a twice-broken beam with several depth planes is shown.

## 5. Model Extensions and Variations

The model as shown above allows for some extensions. For instance, reference [18] shows the simultaneous determination of the refractive index of the liquid by introducing it as an additional unknown into a multimedia bundle adjustment procedure. This may be relevant, if the refractive index is unknown, for instance due to unknown salinity or temperature of the liquid. Both parameters may cause significant changes in the refractive index of water ($∆n_{w}=$ 0.002 per percent salinity, $∆n_{w}=$ 0.00004 per °C in temperature according to (Equation (4)). In an experiment on the validation of refractive index determinability, a standard deviation $\text{σ}n_{w}$ = 0.00015 could be achieved. This value is better than the sensitivity of many optical refractometers. It allows for instance for the determination of the salinity with a standard deviation of less than 0.1%, thus giving the option of examining the properties of the liquid itself in multimedia photogrammetry applications.

**Figure 3:**
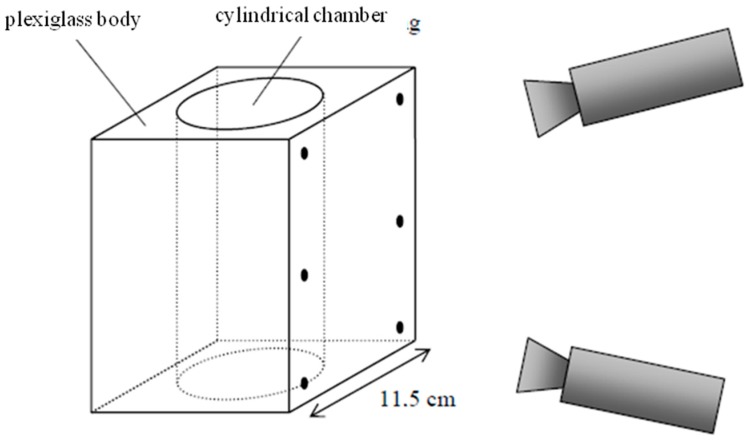
Photogrammetric measurement inside a glass engine [19].

Reference [19] showed an approach to observing phenomena in a plexi-glass combustion engine. The multimedia photogrammetry interfaces herein can be modeled as a plane and a cylinder (Figure 3). The parameters of both interfaces are introduced as unknowns into bundle adjustment, with the adjustment designed as a two-step procedure in order to de-correlate parameters.

Reference [20] showed a sophisticated multimedia photogrammetry model, which is also integrated into a bundle adjustment program and allows for the simultaneous determination of the geometric parameters of an arbitrary number of (not necessarily planar) interfaces in addition to the determination of the refractive index. In an experiment imaging a 3D target field under water with cameras in air through a planar interface, he was able to determine 3D object point coordinates, camera orientation and calibration parameters, planar interface geometry parameters as well as the refractive index simultaneously.

Wolff [21] introduced a new representation and taxonomy of optical systems, wherein the projection center may be a point, a line or a plane, and shows the applicability to multimedia photogrammetry in processing data of an experiment on the photogrammetric reconstruction of fluvial sediment surfaces.

Several authors discuss simplified models of underwater photogrammetry with camera and object under water and the camera viewing perpendicularly to a planar interface. Telem *et al.* [22] avoid the strict modeling of multimedia geometry by absorbing the multimedia photogrammetry effects (which show a radial symmetric behavior if the camera viewing direction is perpendicular to the planar glass interface) by the camera constant and radial lens distortion parameters. Lavest *et al.* [23] state that the effective focal length in underwater photogrammetry is approximately equal to the focal length in air, multiplied by the refractive index of water. Agrafiotis *et al.* [24] extend this model by also considering the dependency on the percentages of air and water within the total camera-to-object distance. Obviously, these models only hold for underwater photogrammetry cases with a camera viewing perpendicularly onto a planar interface.

## 6. Accuracy Aspects

**Figure 4:**
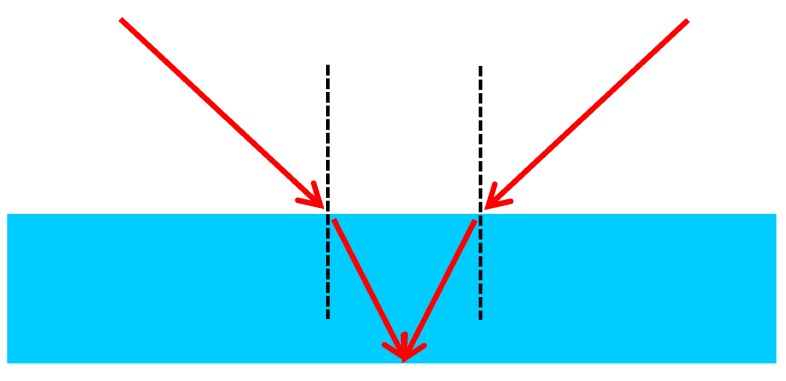
Forward intersection angle in two-media photogrammetry.

The nature of underwater imaging and the necessity of applying geometric multimedia photogrammetry models imply several aspects degrading the accuracy potential of underwater photogrammetry. Therefore, despite strict geometric modeling, the accuracy potential in underwater/multimedia photogrammetry will usually be significantly worse than in conventional photogrammetry. Some important degrading factors are briefly discussed in the following sections:

**Network geometry:** The refraction according to Snell’s Law reduces the opening angle of a camera when viewing from air into water due to the higher refractive index of water. As one can see from Figure 4, the refraction may also lead to a smaller ray intersection angle in 3D coordinate determination from stereo imagery and thus degrade the depth coordinate precision when imaging through the optical media air-(glass)-water [18].

**Interface planarity:** Deviations from planarity in the glass interface between the optical media air and water will lead to variations in the surface normal vectors. This directly translates into errors in the local incidence angles, consequently leading to 3D object coordinate errors. The size of this effect depends on the quality of the glass of the “planar” interface. The effect may be rather large if low quality glass is being used. Simultaneous modeling of the glass interface geometry is rather complex and will often lead to an over-parameterization of the system. Obviously, the effect is much worse when omitting the glass interface and observing objects under water with a camera in air through the spatio-temporally changing wave pattern of an open water surface (which is the standard case in photo bathymetry [6]).

**Refractive index:** Local inhomogeneities of the refractive index of the liquid (for instance due to temperature or salinity gradients within the liquid) will lead to multiply curved optical paths to be handled in photogrammetric tools, which can hardly be modeled. Practical experiments in [11] showed that, while the simultaneous determination of a homogeneous refractive index in multimedia photogrammetry turned out to be possible (cmp. Section 5), the determination of a spatially resolved refractive index field failed due to extremely high correlations in the equation system.

**Figure 5:**
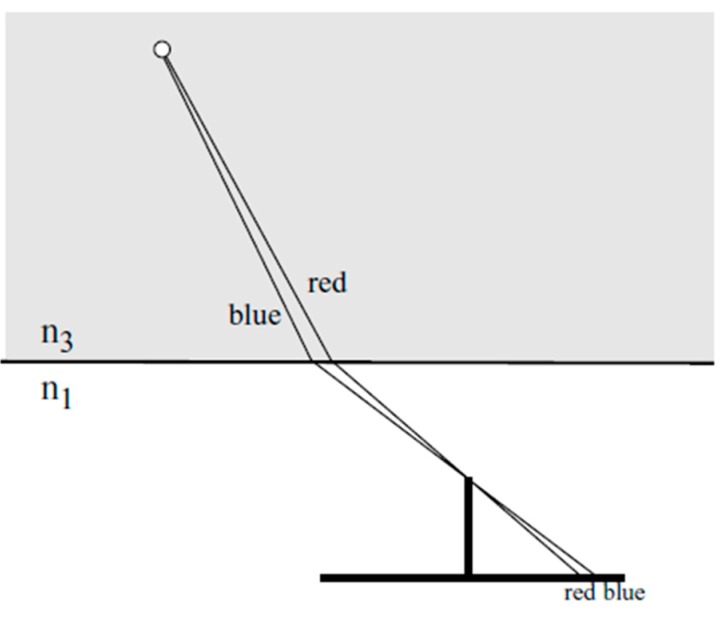
Effect or dispersion [18].

**Dispersion:** The variation of the refractive index over the visible part of the electro-magnetic spectrum is 1.4% in water, while it is only 0.008% in air ([2], cmp. (Equation (4)). Shorter wavelength (blue) light experiences a stronger refraction than longer wavelength (red) light, leading to color seams (red towards the nadir point, blue outward) in RGB images or blur in black-and-white images (Figure 5). These blur effects will reduce the image quality as well as the image measurement precision potential. For standard solid state sensors having a larger sensitivity in the red than in the blue, the effects will even be asymmetric, thus leading to a systematic shift of the centroid of imaged targets. Using Bayer pattern based RGB cameras, interferences between the dispersion effect and the Bayer pattern have to be expected.

Yau *et al.* [25] suggest a model to cope with dispersion effects by handling wavelength-dependent pay paths in the calibration procedure through one or more planar layers perpendicular to the camera viewing direction.

**Diffraction:** Reference [26] has shown, that effects of diffraction cannot be assumed to be symmetric anymore in convergent camera configurations in multimedia close range photogrammetry, leading to a further decrease of image quality and image measurement precision.

**Image focus:** The best focus plane known from conventional photography is not planar anymore when imaging objects underwater. In limited depth-of-focus conditions, this may increase defocusing effects.

**Lens design:** As long as standard camera lenses are used, they will (especially in a convergent configuration) not be optimized for the optical system air-glass-water, again leading to a degradation of image quality and image measurement precision.

**Water quality:** Turbidity, small particles and gas bubbles in the water will cause absorption and diffusion effects, thus reducing image brightness and contrast. Especially in larger water depth and unfavorable turbidity conditions, this will also contribute to an impaired measurability of image coordinates [2].

## 7. Loss-of-Accuracy Validation

**Figure 6:**
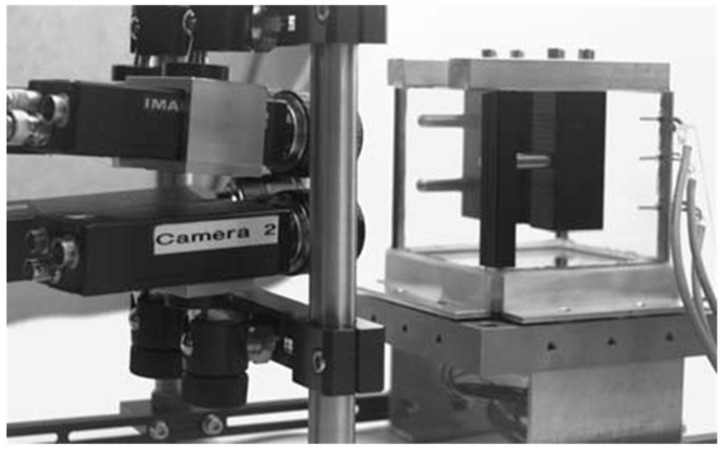
4-Camera system in thermo-capillar convection experiment [27].

To show the accuracy degradation effects discussed in the previous section, some small experiments were conducted imaging a calibration target reference field used for the calibration of a photogrammetric system designed for thermo-capillar convection flow velocity field determination [27]. The 200 × 150 mm^2^ calibration field was placed into the experimental cell made of graded glass. It was imaged by a black-and-white four-camera arrangement (Figure 6) first without water in the glass vessel (*i.e.*, optical path air-glass-air) and then with water in the vessel (*i.e.*, optical path air-glass-water). Between the two experiments, the camera settings remained unchanged; only the orientation angles ω and φ had to be re-adjusted in order to warrant an identical field of view in both experiments. Data processing was performed introducing some of the reference targets as control points and 39 targets as (unknown) check points for an external precision check.

The following three bundle adjustment computations were performed:

- Processing of the air-glass-air case, camera orientation and calibration parameters introduced as unknowns.
- Processing of the air-glass-water case, camera orientation parameters introduced as unknowns, camera calibration parameters taken from I (as camera settings were unchanged).
- Processing of the air-glass-water case, camera orientation and calibration parameters introduced as unknowns.

**Table 1:** Results from multimedia photogrammetry validation experiment.

	${\hat{\partial }}_{0}$ (bundle)	Internal Object Point Precision ${\hat{\partial }}_{XYZ}$	External Object Point Precision $\mu_{XYZ}$
I	0.49 μm	0.010/0.011/0.023 mm	0.013/0.011/0.024 mm
II	1.96 μm		0.031/0.072/0.153 mm
III	1.10 μm		0.021/0.034/0.044 mm

The following conclusions can be drawn from the results of the experiment as shown in Table 1:

- The results in (I) are according to the expectations: The standard deviation of unit weight obtained from the bundle adjustment is in the order of ^1^/_25_ pixel, and the rather good congruence between the internal 3D object point precision parameters (obtained from the self-calibrating bundle adjustment) and the external precision parameters (obtained from 39 independent check points) proves the absence of errors in the geometric and stochastic model.
- In (II) with water filled into the vessel and the cameras re-oriented to capture the same field of view, the external precision figures are much worse (approximately by a factor 5) than in (I). This has to be contributed to the aspects discussed in the former section (except the planarity of the glass interface, as this was present in both experiments).
- In (III) with another self-calibration performed, results get significantly better than in (II) and are only by approximately a factor 2 worse than in (I). This can be explained by the fact that some of the effects discussed in Section 6 show a systematic nature, and that these effects are at least partly compensated by the camera self-calibration parameters. In fact, a significance test between the two parameter sets yielded a highly significant difference between the camera calibration parameters obtained in air and water, despite un-changed camera settings. The largest difference was found in the image shear parameter, which is thus taking a large amount of the systematic part of the errors introduced by the effects discussed in Section 6.

As a conclusion, one can state that the experiment proves the degradation of the accuracy in a multimedia environment. One can also see, that a self-calibration of the cameras in the actual environment leads to better results than pre-calibrated cameras, because errors coming from the multimedia environment show a partly systematic behavior and are partly compensated by camera calibration parameters. Although the conditions in this experiment were rather favorable (low depth, clear water, uniform temperature, zero salinity), the degradation of the geometric precision still amounts to approximately a factor two, with much stronger degradations to be expected under less favorable conditions.

These results correspond to results published in the literature. For instance, Menna *et al.* [28] also report a loss of precision (based on internal bundle adjustment standard deviations) by a factor two, with the largest loss in depth direction. Similar degradations will also have to be faced in other optical 3D underwater measurement techniques. For instance, Ekkel *et al.* [29] report a degradation (also under rather favorable conditions) of the accuracy of profile measurements with a laser triangulation system from 22 μm in air to 35 μm in sweet water. They report a further significant degradation of accuracy when applying the system in salt water.

## 8. Conclusions and Outlook

The paper has shown that strict geometric modeling in underwater and multimedia photogrammetry is possible by a flexible geometric model based on virtual underwater 3D points obtained by a nadir point and depth dependent radial shift of target points. The model depicts an elegant solution, which can be introduced as a module to strictly model ray paths in standard photogrammetric tasks such as spatial intersection, multiple-image forward intersection and self-calibrating bundle adjustment. It can be extended, for instance towards the simultaneous determination of the refractive index. Users of widely-used off-the-shelf photogrammetry software packages such as structure-from-motion tools, which are also becoming widely used in underwater photogrammetry for archaeology and ecology surveys, have to keep in mind that neglecting the effects caused by refraction in the imaging process (or trying to absorb it by standard lens distortion compensation parameters) contributes to a degradation of the quality of results.

Even with strict geometric modeling, underwater/multimedia photogrammetry is accompanied by some effects, which lead to a degradation of the accuracy potential of photogrammetric underwater 3D measurements. Although these effects are partly compensated by camera self-calibration parameters, a degradation by approximately a factor two was obtained in an experiment with rather controlled and favorable conditions. More research has to be performed to further reduce these degrading effects by further improved geometric modeling and adapted self-calibration schemes.
